# Transcriptome Analysis of Drought-Resistant and Drought-Sensitive Sorghum (*Sorghum bicolor*) Genotypes in Response to PEG-Induced Drought Stress

**DOI:** 10.3390/ijms21030772

**Published:** 2020-01-24

**Authors:** Salah E. Abdel-Ghany, Fahad Ullah, Asa Ben-Hur, Anireddy S. N. Reddy

**Affiliations:** 1 Department of Biology and Cell and Molecular Biology Program, Colorado State University, Fort Collins, CO 80523, USA; Salah.Abdel-Ghany@colostate.edu; 2 Computer Science Department, Colorado State University, Fort Collins, CO 80523, USA; fahadullahkhattak@gmail.com (F.U.); asa@cs.colostate.edu (A.B.-H.)

**Keywords:** sorghum, transcriptome, polyethylene glycol (PEG), drought, gene expression, drought resistance

## Abstract

Drought is a major limiting factor of crop yields. In response to drought, plants reprogram their gene expression, which ultimately regulates a multitude of biochemical and physiological processes. The timing of this reprogramming and the nature of the drought-regulated genes in different genotypes are thought to confer differential tolerance to drought stress. Sorghum is a highly drought-tolerant crop and has been increasingly used as a model cereal to identify genes that confer tolerance. Also, there is considerable natural variation in resistance to drought in different sorghum genotypes. Here, we evaluated drought resistance in four genotypes to polyethylene glycol (PEG)-induced drought stress at the seedling stage and performed transcriptome analysis in seedlings of sorghum genotypes that are either drought-resistant or drought-sensitive to identify drought-regulated changes in gene expression that are unique to drought-resistant genotypes of sorghum. Our analysis revealed that about 180 genes are differentially regulated in response to drought stress only in drought-resistant genotypes and most of these (over 70%) are up-regulated in response to drought. Among these, about 70 genes are novel with no known function and the remaining are transcription factors, signaling and stress-related proteins implicated in drought tolerance in other crops. This study revealed a set of drought-regulated genes, including many genes encoding uncharacterized proteins that are associated with drought tolerance at the seedling stage.

## 1. Introduction

Drought is one of the most important abiotic stresses that adversely affects plant growth and productivity (reviewed in [1]). It is the most common cause of food shortage in many countries [2] where it has caused over $29 billion in losses between 2005 and 2015 [3]. Plants use three main strategies (drought escape, drought avoidance, and drought tolerance) to survive under drought stress (reviewed in [4]). To cope with drought stress, plants reprogram a wide range of responses at the molecular, biochemical and physiological levels [5]. These changes often occur rapidly and with specificity based on the tissue type, developmental stage, and stress condition. At the molecular level, the response to drought stress includes transcriptional and post-transcriptional regulation of gene expression [1,6,7]. Transcriptional modulations lead to the differential expression of genes that function in multiple metabolic pathways, which in turn lead to changes in the flow of metabolites and physiological changes associated with protection from cellular damage [8,9,10]. Some of the stress-responsive transcription factors (TFs) are induced very quickly (within minutes) and often transiently while others are activated by stress more slowly (within hours) [11]. Previous studies have shown that the expression of most studied dehydration-responsive element binding (DREB) proteins is low or very low in the absence of stress and is induced moderately under stress and in some cases such as AtDREB2A the activity is enhanced further by stress-induced post-translational activation [10,12]. 

Sorghum (*Sorghum bicolor* (L.) Moench), a C_4_ crop plant used for food, feed, and fiber, is one of the best-adapted cereals to water-limited environments and ranks amongst the most drought-tolerant of all crops grown in the US [13]. Its tolerance to drought is a consequence of heritable morphological, and anatomical characteristics (such as thick leaf wax and deep root system), physiological responses (such as osmotic adjustment and stay green trait) and adaptive mechanisms that allow tolerance under extreme drought conditions (reviewed in [14]). Sorghum cultivars and hybrids that are used today for high grain yield, early flowering, adaptation to drought and bioenergy were selected by farmers and plant breeders from the five sorghum taxa that arose after the divergence of sorghum from rice ~50 million years ago in different regions in the Sahara Desert in Africa [15,16,17,18]. 

Drought resistance, like many agriculturally important traits, is controlled by many genes [19,20,21,22]. The understanding of the mechanisms that contribute to drought stress resistance in sorghum is limited. Several quantitative trait loci (QTLs) that contribute to drought tolerance in sorghum have been identified [20,21,22,23]. However, genes in most of these QTLs that contribute to drought resistance have not been identified [20]. Some drought-related genes in QTLs encode important regulatory proteins and enzymes that are related to drought stress [20]. The availability of the sorghum genome sequences [24,25] and global gene expression studies using either microarray [26] or RNA-Seq [27,28] are uncovering genes and gene networks that contribute to the stress tolerance of sorghum. Transcriptome analysis of sorghum root and shoot treated with polyethylene glycol or abscisic acid revealed over 5000 differentially expressed (DE) genes [27] and the genes coding for late embryogenesis abundant (LEA) proteins, water stress-induced protein (WSI18) and dehydrins were the top up-regulated ones. In another transcriptome study by probing microarrays with cDNA from heat, drought and combined heat and drought stresses Johnson et al., [26] identified sets of genes, which are differentially expressed in response to each treatment, and set of genes that are differentially expressed (DE) only in response to combined stresses. In a recent transcriptome study of two sorghum genotypes with contrasting water use efficiency (WUE) in response to drought Fracasso et al., [28] observed that higher number of DE genes in the sensitive genotype and highlighted the differences between the two genotypes in coping with the drought stress and the strategies adopted. Drought-responsive genes have been reported in other monocots also [29,30,31,32].

To develop drought-resilient sorghum lines either through genetic engineering or molecular breeding it is necessary to identify genes whose expression is associated with drought resistance about which little is known. In sorghum, there is considerable natural variation in drought tolerance [20]. The availability of drought-tolerant and susceptible cultivars at different developmental stages (seedling, pre-flowering, and post-flowering) facilitates global analysis of gene expression to identify drought-regulated genes only in tolerant genotypes, which will then pave the way for developing drought-tolerant crop plants. Therefore, the goal of this study is to exploit natural variation in drought tolerance among genotypes to identify genes that are associated with drought tolerance at the seedling stage. To accomplish this, we performed a transcriptomic analysis of gene expression in two drought-resistant and two drought-sensitive sorghum cultivars in response to polyethylene glycol (PEG)-induced drought stress in aqueous cultures. Although PEG has been widely used to simulate drought stress [4,33,34,35] other soil-based approaches involving the withholding of water gradually or completely stopping of watering plants, and PEG-infused agar plates have also been used [4]. This study expands on the previous sorghum transcriptome studies [26,27,28] by analyzing the expression of all annotated sorghum genes [36] and identifying genes that are associated with drought resistance at the seedling stage. Our study uncovered a set of genes that are expressed only in drought-resistant cultivars. These include many genes of unknown function and others that were implicated in drought tolerance in other plants. 

## 2. Results and Discussion

### 2.1. Selection of Drought Resistance Sorghum Genotypes at the Seedling Stage

**Figure 1:**
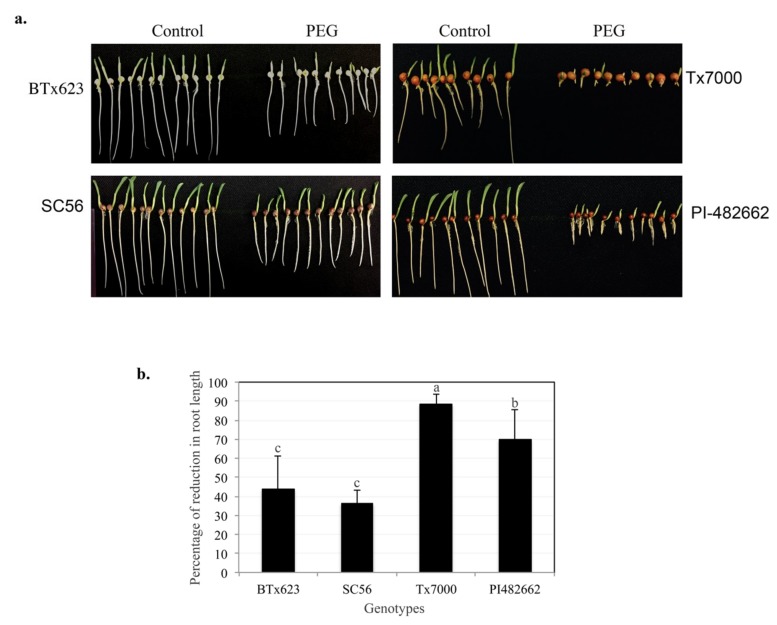
Evaluation of drought tolerance in four sorghum genotypes. (**a**) Seeds of four genotypes (BTx623, SC56, Tx-7000, PI-482662) were germinated and grown vertically on MS (control) and MS-containing polyethylene glycol (PEG) plates. (**b**) Quantification of reduction in root length in four genotypes under drought stress. The root length was measured after 48 h using ImageJ. Percent reduction in root length in the presence of PEG as compared to control is presented. Three biological replicates were used for each genotype. Tukey-Kramer HSD test was performed and significant differences (*p* < 0.05) between genotypes are labeled with different letters. The error bars represent +SD.

To screen for drought resistance at the seedling stage the root length of seven sorghum genotypes was evaluated in the presence and absence of 20% PEG, which has been extensively used to induce water deficit in plants in a controlled manner [4,33,34,35]. The data for the two most drought-resistant genotypes (BTx623 [DR1] and SC56 [DR2]) and the two most drought-sensitive genotypes (Tx-7000 [DS1] and PI-482662 [DS2]) are presented in Figure 1. The genotypes DR1 and DR2 showed the least reduction in root length (Figure 1). On the other hand, genotypes DS1 and DS2 showed the most reduction in root length (88% and 70%, respectively) compared to seedlings grown under control conditions (Figure 1b). Both the BTx623 and SC56 genotypes are grown for grain. BTx623, a complex taxon originated from BTx3197 and SC170-6, is pre-flowering drought-resistant but drought-sensitive at the post-flowering stage (Supplementary Table S1). SC56, a *Caudatum-nigricans* from Sudan, is post-flowering drought tolerant. Tx-7000 (Caprock) is a high yield elite line used for grain production in the US since the 1940s and was used in breeding programs for the production of sorghum hybrids. Tx-7000 is pre-flowering and pre-anthesis drought tolerant while it is susceptible to post-flowering drought stress. PI-482662 (Kafir), originated in Zimbabwe, is grown for beer and is drought tolerant at the later stage of development (Supplementary Table S1). 

### 2.2. Transcriptome Analysis of Two Drought-Resistant and Two Drought-Sensitive Sorghum Genotypes in Response to PEG-Induced Drought

To identify genes that are differentially expressed (DE) in drought-resistant versus drought-sensitive sorghum genotypes in response to drought we carried out RNA-seq analysis using Illumina HiSeq 2000. Drought stress was applied to 8-day-old seedlings by growing them in 20% PEG for 1 and 6 h and the untreated seedlings were used as controls. We have chosen 1 and 6 h time points for two reasons. First, our interest was to investigate rapid changes in gene expression in response to drought stress. Second, we and others have previously shown that drought marker genes are induced within 1 to 6 h [32,37] Two biological replicates were analyzed for each condition resulting in 32 samples (4 genotypes × 2 conditions × 2 time points × 2 replicates). The correlation coefficient between biological replicates was high (*R*^2^ = 0.87–0.99) supporting the reproducibility of the results. In total, about 110–210 million reads, each 50 nucleotides long, were generated for each sample. These reads were aligned to the reference genome and approximately 85–90% of the reads were aligned to the reference genome (Table 1). About 90–96% of these mapped reads were aligned to only one position in the reference genome while the rest were aligned to more than one position (Table 1). The assembled transcripts were mapped to in-house assembled sorghum transcriptome (assembled from *Sorghum bicolor* v3.1 DOE-JGI, (http://phytozome.jgi.doe.gov/) and [36]). 

**Figure 2:**
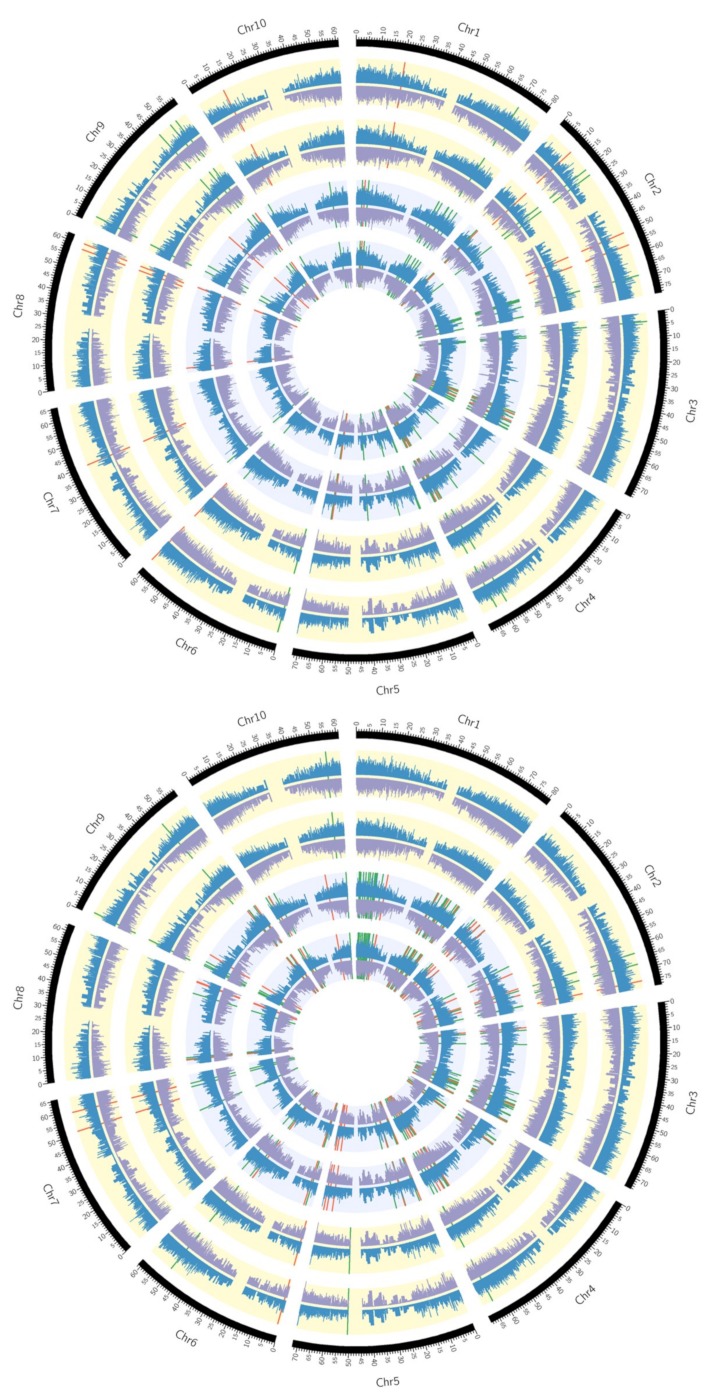
Genome-wide differential gene expression in the drought-resistant (DR1 and DR2) and sensitive (DS1 and DS2) lines of sorghum at 1 h (top) and 6 h (bottom). Each slice represents one of the 10 sorghum chromosomes. From the center, the first four concentric circles represent sorghum lines DR1, DR2, DS1, and DS2 respectively. The outermost circle shows the genomic coordinates with a step size of 10 million bp. The gene expression levels are shown by blue and purple coverage plots for the control and treated samples, respectively. Across the coverage plots, the up-regulated and down-regulated genes unique to resistant (DR1 and DR2) and sensitive (DS1 and DS2) genotypes are marked by green and red lines, respectively.

**Figure 3:**
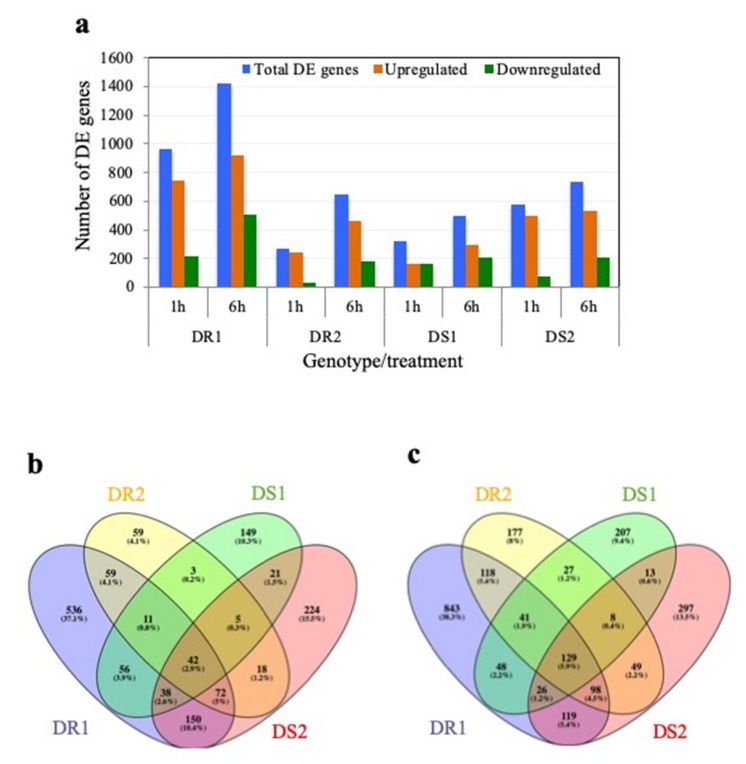
Differentially expressed (DE) genes in different genotypes in response to drought. (**a**) Total number of DE, up- and down-regulated genes that passed the cut-off Log_2_ FC > 2 and q-value < 0.05 as compared to control condition. (**b**,**c**) Venn diagrams showing the unique and common DE genes among genotypes at 1 h (**b**) and 6 h (**c**).

The transcript abundance of each gene was calculated as fragments per kilobase per million mapped reads (FPKM) and these values were then used to determine the DE as log_2_ fold change (FC) ratio between the control and the treated for each time point and in each genotype. After applying the cut-off log_2_FC ≥2 for up-regulated and ≤−2 for down-regulated and the corrected *q*-value cut-off <0.05 the differentially expressed (DE) genes were identified. Figure 2 shows up- and down-regulated genes at 1 and 6 h, respectively, on each chromosome in all four genotypes in response to PEG-induced drought stress. The total number of DE genes was very different among tested genotypes and at different treatment times with the highest number observed for the DR1 genotype at both 1 and 6 h treatments (954 and 1319 genes, respectively) (Figure 3a). Also, the total number of DE genes was higher at 6 h treatment than at 1 h treatment in all tested genotypes. Among the DE genes, the number of up-regulated ones was higher than the down-regulated ones in all tested genotypes especially after 1 h treatment (Figure 3a) indicating that the sorghum genotypes differ in their response to drought stress and the drought response mostly involves up-regulation of specific sets of drought-responsive genes. 

Next, we explored the unique as well as the common differentially expressed genes in different genotypes in response to drought stress (see Figure 3b,c). There is a unique set of DE genes for each genotype as well as common DE genes among genotypes at each time point. Forty-two and 129 common drought stress-responsive genes were differentially expressed in all lines at 1 and 6 h, respectively (Figure 3b,c). About 35% of these DEGs are considered “not annotated”. Interestingly, all 42 and about 95% of the 129 genes were up-regulated (Supplementary Table S2, sheets A and B), suggesting that drought response in sorghum mainly involves up-regulation of a set of stress-responsive genes. Metabolism overview and stress mapping using the MapMan software showed that these genes encode transcription factors, and other proteins involved in hormone signaling, secondary metabolism, stress, detoxification/antioxidant as well as proteins of unknown function (Supplementary Figure S1a,b). Comparison between the early (1 h) and late (6 h) stress response revealed that genes involved in auxin, ABA and JA signaling and genes encoding AP2/ERF, DREBs and MYB transcription factors (TFs) are amongst the early response genes, while after 6 h treatment genes involved in abiotic stress, secondary metabolism, heat shock, and glutathione-S-transferases synthesis are the most enriched ones (Supplementary Figure S1c,d). DREBs/CBFs are amongst the first families of TFs that are induced by water deficit [10] to regulate the expression of downstream stress-responsive genes [38]. The expression of sorghum DREB2 (SbDREB2) in rice under the control of stress-inducible *rd29A* promoter was induced at 1 h exposure to drought, after which expression gradually dropped to basal level [39]. Only 16 genes were differentially expressed in the four genotypes at both time points (Supplementary Table S2, sheet G). Amongst the most highly elevated transcripts are those encoding water stress-induced protein 18 (WSI18), alpha-amylase and glutathione-S-transferase which are known to be involved in general water deficit signaling and tolerance. Interestingly, all DE genes in all genotypes at both time points are significantly up-regulated except C-terminal thiamine pyrophosphate binding domain-containing protein (*SOBIC.009G16900*) which is down-regulated in DR2 and DS1 at 6 h PEG treatment (Supplementary Table S2, sheet G). Many of the DE genes expressed only in resistant lines (e.g., LEA genes, transcription factors, proteins involved in signaling and lipid metabolism) were also reported to be drought-regulated in sorghum plants subjected to drought by withholding water [26,28]. 

### 2.3. Differentially Expressed Genes Associated with Drought Resistance

**Figure 4:**
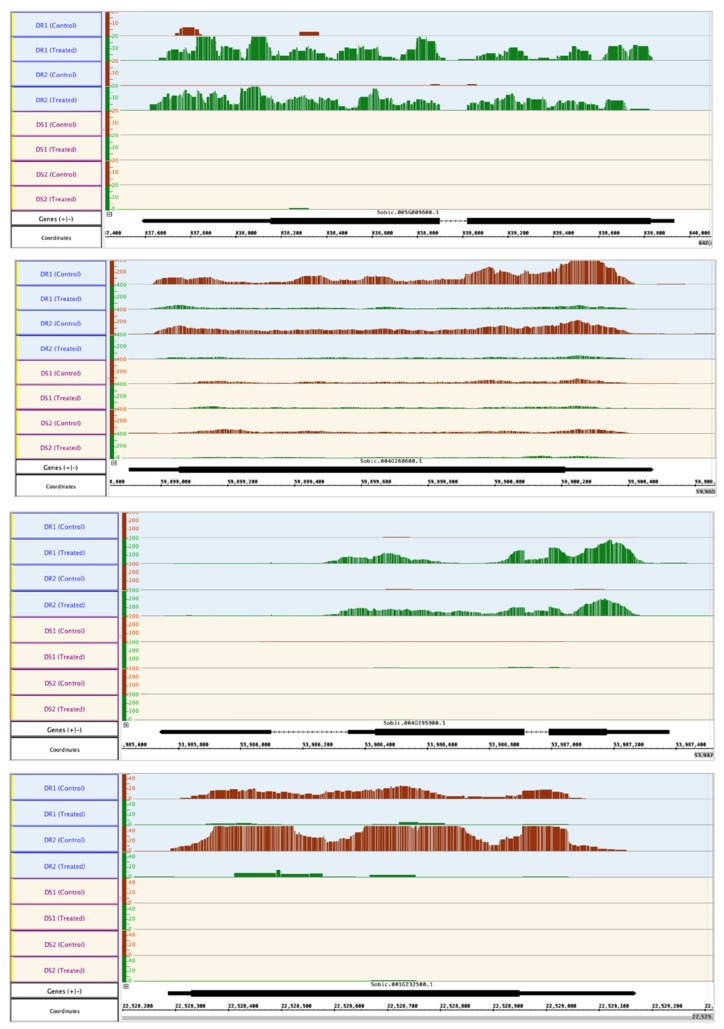
Relative sequence read abundance (Integrated Genome Browser view) as histograms of differentially expressed genes only in the drought-resistant (DR1 and DR2) genotypes. Top two panels) One up-regulated (top) and one down-regulated gene (bottom) in resistant genotypes (DR1 and DR2) after 1-hr PEG-treatment. Replicates of each line are shown. Bottom two panels) One up-regulated (top) and one down-regulated gene (bottom) in resistant genotypes (DR1 and DR2) after 6-hr PEG-treatment. Replicates of each line are shown. The Y-axis indicates the read count. Gene structure and gene ID are shown below the read tracks. In the gene structure, lines represent introns and the boxes represent exons. The thinner boxes represent 5′ and 3′ UTRs.

Previous studies concerning the mechanism of abiotic stress tolerance in sorghum have focused on either one genotype [26,27,40] or two genotypes with differences in drought tolerance at the pre-flowering stage [28]. Here, we have compared two drought-resistant (DR1 and DR2) and two drought-sensitive genotypes (DS1 and DS2) at two time points (1 and 6 h PEG post-treatment) to identify the early and late response genes that might participate in drought resistance of sorghum at the seedling stage. Fifty-nine and 118 of the DEG were common between the two drought-resistant genotypes at 1 and 6 h, respectively (Figure 3b,c, Table 2 and Table 3) compared to 21 and 13 DEG in drought-sensitive genotypes at 1 and 6 h, respectively (Figure 3b,c, and Supplementary Table S2, sheets E and F, respectively). The location of up- and down-regulated genes on sorghum chromosomes at each time point is shown in Figure 2. An Integrated Genome Browser view showing the read abundance of one up-regulated and one down-regulated gene in resistance genotypes at each time point is shown in Figure 4. 

**Figure 5:**
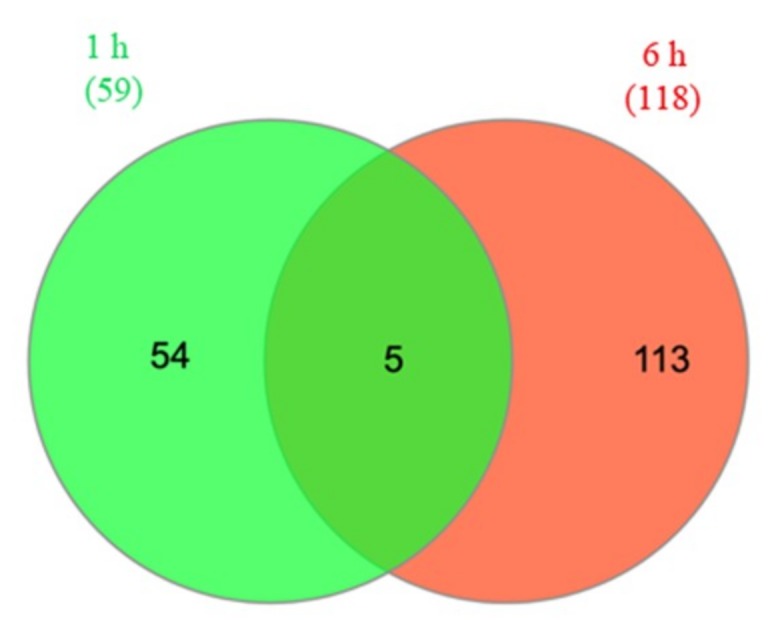
Venn diagram showing the overlap between genes differentially expressed only in drought-resistant genotypes (DR1 and DR2) genotypes at 1 and 6 h.

**Figure 6:**
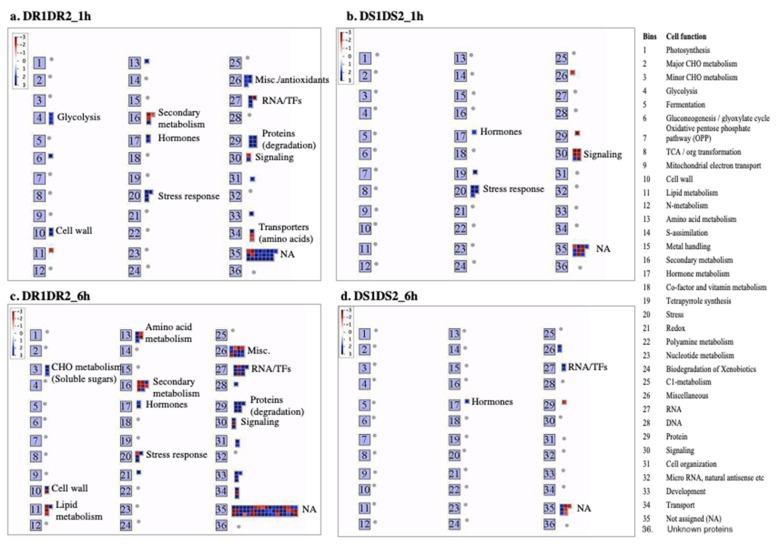
Functional categories of DE genes expressed only in drought-resistant (DR1 and DR2) and drought-sensitive (DS1 and DS2) sorghum genotypes at 1 h (**a**,**b**) and 6 h (**c**,**d**) of post PEG-treatment. Blue represents up-regulated and red represents down-regulated genes. This figure was generated using MapMan and show DE genes that passed the cut-off value of Log_2_ FC ≥2 and ≤−2 and *q*-value < 0.05. NA, Not Assigned.

There is very little overlap between drought-regulated genes in drought-resistant genotypes between the 1 h and 6 h time points (Figure 5). Of the five common DE genes at both time points, two of them are uncharacterized proteins. The functional category of the DE genes using MapMan software highlighted the early response (1 h) genes and the late response (6 h) genes that might participate in drought responses in the drought-resistant genotypes (Figure 6a–d). These functional categories include genes that encode TFs or proteins involved in protein degradation, metabolism-related proteins (carbohydrates; lipids and secondary metabolites), hormone signaling, stress response, amino acid transport, cell wall in addition to proteins involved in miscellaneous functions such as detoxification/oxidation-reduction/and antioxidants, many of which have been implicated in stress tolerance in other plants. These functional categories can be grouped into two main categories—transcription factors and damage limitation/repair mechanisms. Below, we discussed the role of DE genes that are expressed only in drought-resistant genotypes.

#### 2.3.1. Transcription Factors and Stress Tolerance

Transcription factors play a crucial role in plants’ adaptation to stresses because they act as master switches and regulate the expression of stress-responsive genes [11]. The AP2/EREB subfamily is amongst the TFs that transiently respond to abiotic stress and their constitutive expression causes drought tolerance in many species. Four of these AP2/EREB TFs were induced in response to drought in the drought-resistant genotypes and not in the sensitive ones (Table 2 and Supplementary Figure S1) suggesting that they might be part of the resistance mechanism. The expression level of these TFs increased between four and ten-fold in treated conditions compared to controls in the drought-resistant genotypes. Among these, AP2 domain-containing proteins are two candidates that have similarity to dehydration-responsive element binding proteins (DREB1a and DREB1b), while others have similarity to ethylene-responsive element binding TFs (EREB), both of which are ubiquitous in plants. Interestingly, sorghum *DREB2* expression driven by a stress-inducible promoter in rice improved both tolerance and yield under drought stress [39]. A similar study in tobacco has shown that AP2/ERF TFs were the main group of up-regulated genes after 40 min of drought stress [41]. In addition to the induced AP2 domain-containing TFs, one AP2/ERF TF (*SOBIC.002G071600*) was down-regulated in drought-tolerant cultivars in response to drought (Table 2). This gene product has sequence similarity to RAP2.11 (related to AP2.11) from other plant species. In Arabidopsis, RAP2.11 modulates plant response to low potassium through regulation of the high-affinity K^+^ uptake AtHAk5 transporter and other genes related to low K^+^ signaling including ROS production [42]. 

To test if the expression of the early response AP2/ERF TFs continued for a period of time and what other TFs are induced slowly in response to drought we analyzed the DE genes after 6 h PEG treatment. Interestingly, none of the early response TFs was detected at 6 h (Supplementary Table S2, sheet D and Supplementary Figure S1). However, other TF genes that belong to different families were differentially expressed only in drought-resistant genotypes. These include members of AP2/ERF, bZIP, Zinc finger, bHLH, MYB and GRAS (Supplementary Table S2, sheet D and Supplementary Figure S1). Homologs of these TFs were found to have essential roles in stress tolerance in other plant systems. For example, the homolog of the GRAS/SCL transcription factor that is encoded by *SOBIC.001g174100* is induced in response to drought and its overexpression in Arabidopsis and rice enhanced tolerance to drought via binding to promoter elements in stress-responsive targets [43,44]. Another abiotic stress-related TF that is highly induced in sorghum resistant genotypes at 6 h is A20/AN1 zinc-finger containing stress-associated protein (SAP) encoded by *SOBIC.007g138000*. In rice, this TF interacts with a receptor-like kinase to improve water deficit tolerance via a signaling pathway that affects the expression of several stress-responsive genes including *LEA, rd29A, CBF/DREB* and *MAP* [45]. Also, histone-binding protein-1b (HBP1b), belonging to the bZIP family of TFs, encoded by *SOBIC.009g23000* is induced in sorghum drought-resistant cultivars. In rice, a homolog of this gene is localized within *Saltol* QTL and its expression is differentially regulated in rice seedlings from contrasting genotypes [46]. Transgenic tobacco plants ectopically expressing OsHBP1b showed better survival under drought and salinity [46]. These early and delayed TFs that are differentially expressed in response to drought are mostly responsible for drought stress differences among genotypes. In addition to the annotated TFs, about one-fifth of the DEGs are not annotated and these might encode for other TFs that might also have roles in drought stress resistance. 

#### 2.3.2. Damage Control/Repair and Detoxification

##### Cryo-Protectant

Late Embryogenesis Abundant (LEA) proteins including dehydrins are hydrophilic proteins that have been linked to plant survival under stress. They are induced by abscisic acid and various abiotic stresses such as PEG-induced drought and NaCl. LEA proteins are thought to function in many ways including stabilizers of membranes and proteins and antioxidants to protect cells from damage [47]. In response to water deficit, the expression of these genes is higher in wheat tolerant cultivars compared to sensitive ones [48]. In this study, six out of the top 25 induced genes in resistant genotypes at 6 h (5% of the DEGs) encode LEA proteins in addition to two other dehydrin-type of LEAs (Table 3). Five of these induced LEA proteins have sequence similarities to D-34 and belong to LEA subgroup 5 while the other (LEA 4) belongs to subgroup 3. The expression level of these genes is similar in both drought-resistant genotypes (DR1 and DR2) and ranges between 20 and 400 times based on the absolute fold change (Table 3). A similar result was obtained in another study [26] where they have found that the top 100 DE genes in R16 sorghum genotype in response to drought contains seven LEA genes. Another seed protein, which has a protective role against dehydration, is PM41 seed maturation protein. The expression of this gene is also highly induced in the drought-resistant genotypes (DR1 and DR2).

Heat shock proteins (HSPs) are also induced in response to abiotic stresses. HSP20/alpha crystallin family of HSPs are molecular chaperones that act by avoiding protein denaturation and assist other chaperons in maintaining the native confirmation of nascent polypeptide chains and/or reorganizing denatured proteins to their native forms [49]. The transcripts of two genes encoding HSP20 homologs as well as the HSP101 were also induced in both drought-resistant genotypes at 6 h treatment (Table 3).

Another set of protecting proteins that are induced in response to biotic and abiotic stresses in plants is non-specific lipid transfer proteins (nsLPTs) and fatty acid metabolism-related proteins [50,51]. They may function in the repair of stress-induced damages in membranes or changes in lipid composition/fluidity. In drought-resistant genotypes at 1 h PEG treatment, the transcript of LTPL24 gene (*SOBIC.007g029800*) is highly accumulated even in DR2 genotype it is the highly induced gene (about 700 absolute fold change compared to untreated condition). Lipase is also induced in both genotypes at 6 h treatment. On the other hand, the transcripts of four genes that annotated as LTPL116-protease inhibitor/seed storage/LTP family protein were significantly down-regulated in both drought-resistant genotypes in response to drought stress (Supplementary Table S2). Other fatty acid metabolism-related genes that are regulated in resistant genotypes at both time points are fatty acid desaturase and fatty acid hydroxylases. Down-regulation of these genes suggests that they might perform a different biological role in sorghum in response to drought.

Rare-cold inducible (RCL2) proteins are highly hydrophobic proteins that are also induced in response to water stress in many plant species [52]. They function as membrane protein stabilizers. RCL2-5 (low temperature and salt responsive protein) in rice and Arabidopsis is induced in response to drought and is localized to the plasma membrane suggesting that it might be a membrane protein or as a signal peptide that functions in signal transduction [53]. Eight RCL2 genes are present in the Arabidopsis genome and seven were identified in the rice genome. A homolog of OsRCL 2–5 encoded by *SOBIC.009g025599* is highly induced in drought-resistant genotype especially in DR1 at 6 h treatment where its induction is the highest (about 800 times more compared to untreated condition).

##### Detoxification-Related Genes

Abiotic stresses including drought lead to overproduction of reactive oxygen species (ROS) and methylglyoxal (MG) [54], which ultimately result in oxidative stress. The antioxidants function to protect plants against oxidative stress damages [55]. Interestingly, in drought-resistant genotypes, 4 genes encoding glutathione S-transferases (GSTs) were highly up-regulated at 1 h treatment (8% of the differentially up-regulated genes). At 6 h treatment, no GSTs were among the common up-regulated genes in the drought-resistant genotypes rather there was one gene that is predicted to encode GST (*SOBIC.001g317800*) was down-regulated. Peroxidase and oxidoreductases also participate in ROS scavenging. In addition to GSTs, a gene encoding peroxidase and one gene encoding oxidoreductases were induced in resistant genotypes.

Proteases including cysteine protease and protease inhibitors are also involved in drought stress tolerance in plants [50]. In drought-resistant genotypes, three cysteine proteases EP-B1-encoding genes and one cysteine protease inhibitor gene were among the top 20 up-regulated DEGs at 1 h treatment (Table 2) while none was detected among the annotated DE genes at 6 h suggesting that they are part of the early stress-responsive genes in sorghum drought-resistant genotypes. 

### 2.4. Osmoprotectants

Plants accumulate low molecular weight compounds (osmoprotectants) to cope with drought and salt stresses. Osmoprotectants function to increase the osmotic potential, to protect the proteins and membranes against stress damages and to scavenge the ROS generated under stress [56]. Targeting the engineered osmoprotectant enzymes to the chloroplasts resulted in better stress protection because chloroplasts are the primary site of ROS production [57]. Osmoprotectants are classified into three major groups, which are amino acids, sugars and quaternary amines [58]. In the drought-resistant genotypes (DR1 and DR2) a set of enzymes that are known to participate in drought tolerance in other plant species are up-regulated at 1 and 6 h in response to drought treatment (Table 2 and Table 3). Among these enzymes are alpha-amylase (*SOBIC.004G309000*), isocitrate lyase (*SOBIC.002G324000*), glycosyl hydrolase (*SOBIC.005G099000*), glutamate decarboxylase (*SOBIC.001G443800*), aldose reductase (ALDR) (*SOBIC.009G171400*), galactinol synthase (*SOBIC.001G391300*) and glutamine-dependent asparagine synthetase (*SOBIC.005G003200*). Glutamate decarboxylase (GAD) catalyzes the decarboxylation of glutamic acid to GABA and CO_2_. Several lines of experimental evidence revealed a stress-specific pattern of GABA accumulation in plants, which is consistent with its physiological role in stress mitigation and a signaling role in the perception and transduction of environmental stress signals [59]. Aldose reductase catalyzes the conversion of the harmful MG into osmoprotectant sorbitol and overexpression of xerophytic ALDR in tobacco confers tolerance against salt and drought stresses through detoxification of MG and reducing membrane damage [60]. The products of these enzymes together with other expressed uncharacterized proteins may act as compatible solutes in drought stress resistance and as antioxidants against damage induced by ROS and MG [61].

#### Membrane Composition and Signaling Molecules

Membranes’ lipid composition and lipid-derived signal molecules are among the first cellular components that perceive the drought stress [62]. It appears that changes in membrane lipid metabolism play an essential part in plant responses to different abiotic stresses including drought. Fatty acids desaturases and sterol desaturase are among the most down-regulated genes in drought-resistant genotypes at 1 and 6 h treatments (Table 2 and Table 3). In wheat and other plant species, both salt and drought were found to reduce the amount of 18:3 unsaturated fatty acids [63]. This data suggest that the fluidity of the membrane is important for drought stress tolerance in sorghum.

Inositol 1,4,5-triphosphate (InsP_3_) is a soluble second messenger implicated in plant response to environmental stimuli [64]. Inositol polyphosphate 5-phosphatase specifically hydrolyzes the soluble InsP_3_ [65]. Interestingly, in drought-resistant genotypes, the expression of the inositol polyphosphate 5-phosphatase-like gene is strongly up-regulated in response to PEG treatment at 6 h post-treatment (Table 3). Arabidopsis transgenic plants ectopically expressing a mammalian inositol polyphosphate 5-phosphatase were more drought-tolerant, lost less water and showed less accumulation of ABA compared to wild type plants [65]. Also, the basal level of the drought-inducible ABA-independent transcription factor DREB2A and some of the DREB2A-regulated genes are higher in the unstressed transgenic plants suggesting that InsP_3_ acts as a negative regulator in the DREB2A-mediated drought signaling pathway. In this study, the expression of DREB2A TF and the DREB2A-regulated LEA and dehydrin genes are up-regulated in drought-resistant sorghum genotypes DR1 and DR2 (Table 2).

### 2.5. Motif Analysis in the Promoters of DE Genes

**Figure 7:**
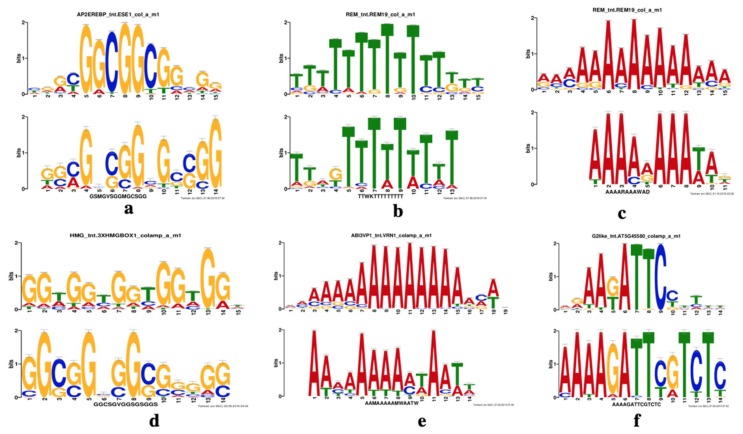
Significant matches in the Arabidopsis TF database are shown for the motifs enriched in the promoter regions of differentially expressed genes in the sorghum drought-tolerant lines (DR1 and DR2) at T = 1 h (**a**–**c**) and T = 6 h (**d**–**f**). The enriched motifs were searched against the Arabidopsis TF database using the TomTom tool from the MEME suite. The transcription factors shown here are (**a**) AP2EREBP (**b**) REM, (**c**) C2C2, (**d**) HMG, (**e**) ABI3VP1, and (**f**) G2like.

**Figure 8:**
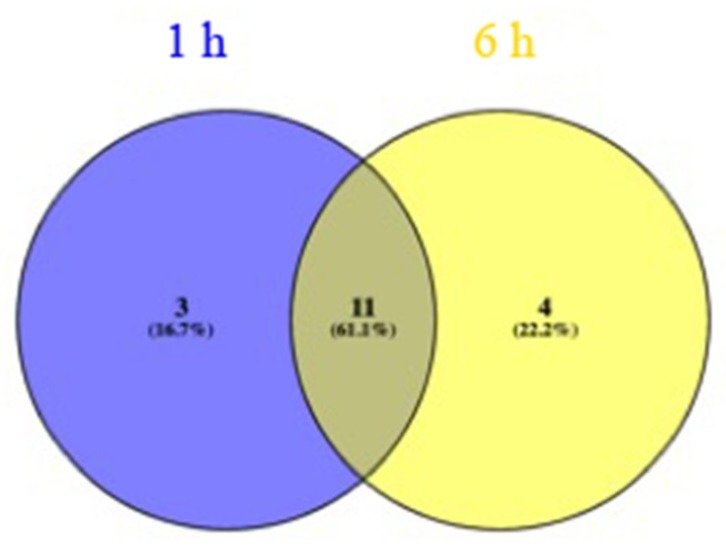
The overlap between transcription factor families significantly matched to the enriched motifs in the promoter regions of the differentially expressed genes at T = 1 h and T= 6 h is shown.

To identify potential *cis*-regulatory elements in the promoters of genes differentially expressed only in drought-resistant genotypes, we searched the promoter sequences (−1 to −1000 bp region) for motifs enriched in DE genes at 1 and 6 h using the MEME suite [66]. The top three enriched motifs in the promoters of DE genes at 1 and 6 h are shown in Figure 7a–c (top panel) and Figure 7d–f (bottom panel), respectively. We then searched these motifs against the binding sites of known transcription factors and identified the closest match. Matches of known TF binding sites for the top three at 1 h and 6 h are shown in Figure 7a–c (top panel) and Figure 7d–f (bottom panel), respectively. Interestingly, some of these TFs are known to be involved in ABA-dependent (e.g., ABI3VP) or ABA-independent (e.g., AP2EREBP family that contains DREB members) drought responses [67,68]. In a previous study, the abscisic acid response element (ABRE) was found to be highly enriched in the promoters of drought-responsive genes [26]. Overall, eighteen TF families matched the motifs found in both 1 and 6 h DE genes. Of these, 3 and 4 TFs are unique to motifs found in 1 h and 6 h DE genes, respectively whereas 11 TF binding sites are found in promoters of DE genes at both time points (Figure 8). The list of these TFs is provided in Supplementary Table S3. The TF binding regions in the promoters of DE genes at 1 h and 6 h are enriched within the −150 bp region (see Supplementary Figures S2 and S3). Together, these results indicate that a specific set of TFs is involved in regulating the expression of genes in response to drought. Further studies such as chromatin immunoprecipitation (ChIP)-Seq with control and drought-treated plants are needed to test the direct binding of these TFs to these promoters. 

It is common in sorghum genotypes to exhibit contrasting drought phenotypes depending on the developmental stage (Table S1) [22,69]. For example, sorghum TX7078 is pre-flowering-tolerant and post-flowering susceptible whereas B35 pre-flowering susceptible and post-flowering-tolerant [69]. Similarly, the four genotypes that we studied here at the seedling stage show contrasting drought phenotypes at seedling, pre- and post-flowering stages (Table S1). This might be due to differential expression of different sets of stress-related genes at different developmental stages thereby conferring contrasting drought phenotypes (tolerance or sensitivity). It would be of interest to conduct drought stress experiments from germination to post-flowering and determine the expression pattern of drought-responsive genes at different developmental stages. In this study, we identified genes associated with drought tolerance/sensitivity at the seedling stage. 

### 2.6. Conclusions

In this study, we identified a set of genes that are differentially expressed only in resistant genotypes in response to PEG-induced drought. This set of genes encodes known transcription factors and other proteins (signaling and metabolic enzymes) that are implicated in drought responses and also many novel proteins with no known function. Bioinformatics analysis indicated potential regulatory elements in these genes and cognate transcription factors that may modulate the expression of these genes. Future functional studies with the uncharacterized genes that are expressed only in the drought-resistant genotypes will further our understanding of drought tolerance in sorghum at the seedling stage and help in developing drought-resistant crops. Increased expression of rapidly induced transcription factors identified in drought-resistant genotypes in drought-sensitive genotypes through genetic engineering approaches could lead to the generation of drought-resistant cultivars.

## 3. Materials and Methods 

### 3.1. Plant Materials and Growth Condition

Seeds of *Sorghum bicolor* genotypes (BTx623, SC56 and Tx7000) were obtained fromCourtney Jahn, Department of Bioagricultural Sciences and Pest Management at Colorado State University and other genotypes (PI-494559, PI-494558, PI-494560, and PI-482662) were obtained from United States Department of Agriculture (USDA). Seeds of the seven cultivars were propagated in a greenhouse at 16/8 h light/dark cycle, 26 °C and light intensity 100–120 microeinstein·m^−2^·s^−1^.

To evaluate the drought tolerance of these genotypes at the seedling stage we compared the root length of seedlings grown vertically on MS and MS-containing polyethylene glycol (PEG)-8000 (Sigma, St. Louis, MO, USA) agar plates with water potential of –0.5 MPa according to [70]. Drought tolerance experiments were conducted four times. In each experiment, three plates with 12 seedlings per plate were used. The root length was measured after 48 h using ImageJ [71]. Two genotypes (BTx623 (DR1) and SC56 (DR2) were selected as drought-resistant (DR) while Tx-7000 (DS1) and PI-482662 (DS2) lines were chosen as drought-sensitive (DS) at the seedling stage. For brevity, the selected cultivars were referred to as DR1, DR2 and DS1 and DS2, respectively.

For drought treatment, seeds of the selected four genotypes were surface sterilized with 20% bleach, rinsed with distilled water, and germinated on wet filter paper for 24 h. The seedlings were then transferred to culture tubes (two seedlings per tube) and allowed to grow vertically on 0.5× Hoagland nutrient solution-wetted 3 M filter paper bridge for an additional 7 days as described [36]. On the 8th day, the solution was completely decanted and replaced with 5 mL of 0.5× Hoagland solution (control) or 5 mL of 20% PEG dissolved in 0.5× Hoagland solution (drought treatment) [27,33]. Seedlings, excluding the seed remnant, were harvested after 1 and 6 h post-treatment, rinsed with cold distilled water, flash-frozen in liquid nitrogen and stored at −80 °C until used for RNA extraction.

### 3.2. RNA Extraction and Library Preparation

Total RNA was extracted using miRNeasy Mini Kit (Qiagen, USA#217004, Germantown, MD, USA) and treated with DNase I using in-column DNase I digestion as described in the user’s manual. The integrity of RNA was checked using Bioanalyzer (Agilent Technologies, Santa Clara, CA, USA) and RNA samples with RNA integrity number (RIN) ~9.0 or more were used for further analyses. Poly (A) mRNA enrichment and construction of stranded mRNA-seq libraries were performed using KAPA Stranded mRNA-seq kit (Cat# KR0960) (Illumina, San Diego, CA, USA). Briefly, poly (A) mRNA was captured using magnetic oligo (dT) beads and fragmented using heat and magnesium. First-strand cDNA was synthesized using random primer and the second strand was synthesized in the presence of dUTP. Adenosine was added to the 3′-end followed by adaptor ligation. The library was amplified using high fidelity, low-bias PCR. Single-end sequencing of the libraries was done at the Sequencing and Analysis Core Resource, Duke University using Illumina Hi seq 2000 (Illumina, San Diego, CA, USA).

### 3.3. Data Pre-Processing

The RNA-seq reads generated by Illumina sequencing were first analyzed for quality control using FastQC (https://www.bioinformatics.babraham.ac.uk/projects/fastqc/). To get rid of adapter sequences and other artifacts, fastx-trimmer (from FASTX-toolkit at http://hannonlab.cshl.edu/fastx_toolkit) was used to trim the first 10 and last 3 bp from each read, respectively. After filtering, the remaining reads were aligned to the *Sorghum* bicolor version 3 reference genome using TopHat2 [72] with the following parameters: -i 10, -I 10000, --b2-sensitive, --segment-length 20. Next, in each library, reads that aligned to multiple locations were filtered out. Uniquely aligned reads were used to analyze differential gene expression. We used SpliceGrapher [73] (script: sam_filter.py) to filter out spurious splice junctions and to predict splice graphs from the RNAseq data. For intra- and inter-genic mapping statistics, we used RSeQC [74] (version 2.6.1). All RNA-seq reads are deposited at NCBI (BioProject ID: PRJNA585370). 

### 3.4. Differential Gene Expression Analysis

To assess replicate reproducibility (same- and cross-condition replicate correlations) we used Cufflinks [75] to compute FPKM per gene. For visualization, we used a modified version of a publicly available python script (https://lingfeiwu1.gitbooks.io/data-mining-in-social-science/content/python_for_data_analysis/). For protein-coding genes, EdgeR [76] was used to quantify differential gene expression across all lines and time points. Note that EdgeR *p*-values were applied with *q*-value adjustment using the Benjamini–Hochberg method. Moreover, we used the log fold change value of 2 (logFC_2_) as the threshold for differential gene expression. 

### 3.5. Circos Plots

Circos [77] plots were generated with FPKM values of genes, in the four main tracks, measured using the Cufflink tool. The differentially expressed genes unique to the resistant and sensitive genotypes were shown in their corresponding tracks.

### 3.6. Motif Analysis

Promoters were defined as 1000 bp regions upstream of the transcription start site of the up- and down-regulated genes. Next, the DNA sequences were extracted from the identified promoter regions. For motif discovery, MEME suite [66] was used in classic mode with the following parameters: maximum number of motifs = 12, minimum motif width = 6, maximum motif width = 14, and background model set to 1st order (adjusted for dimer biases). The statistically significant motifs were searched against the Arabidopsis DAP motifs and Eukaryote DNA motifs databases using the TomTom tool from MEME suite with the following parameters: E-value threshold <10, and motif column comparison function set to Pearson correlation coefficient. For the positional preferences of known motifs (from the Eukaryote DNA and Arabidopsis DAP databases) within the promoter region sequences, we used the CentriMo tool from the MEME suite. The motif enrichment search was set to anywhere within the promoter sequence. The CentriMo tool was used with default parameters such as match score threshold ≥5, and E-value threshold for reporting enriched regions ≤10.

## Figures and Tables

**Table 1:** Alignment statistics of RNA-seq reads to the reference genome.

Genotype	Total Reads (×10^6^)				Mapped Reads (×10^6^)				Uniquely Mapped Reads (×10^6^)				Reads Mapped to Multiple Locatios (×10^6^)			
	1 h		6 h		1 h		6 h		1 h		6 h		1 h		6 h	
	Cont	PEG	Cont	PEG	Cont	PEG	Cont	PEG	Cont	PEG	Cont	PEG	Cont	PEG	Cont	PEG
BTx623 (DR1)	127.0	125.6	130.3	130.9	108.7 85.6%	114.4 91.1%	117.0 89.8%	115.3 88.1%	103.8 95.5%	107.2 93.7%	111.6 95.4%	108.5 94.1%	4.9 4.5%	7.2 6.3%	5.4 4.6%	5.4 5.9%
SC56 (DR2)	129.3	140.7	210.5	174.7	117.1 90.5%	126.1 89.6%	180.2 85.6%	149.7 85.7%	103.1 88.1%	114.4 90.8%	172.1 95.5%	136.8 91.3%	13.9 11.9%	11.6 9.2%	8.2 4.5%	13.0 8.7%
Tx-7000 (DS1)	131.6	123.4	126.3	137.1	117.3 89.1%	109.1 88.4%	111.1 87.9%	119.7 87.3%	108.6 92.6%	104.4 95.7%	106.3 97.7%	112.2 93.8%	8.7 7.4%	4.7 4.3%	4.7 4.3%	7.4 6.2%
PI482662 (DS2)	115.4	109.8	122.8	110.9	100.5 87.0%	94.9 86.4%	107.3 87.4%	94.1 84.8%	95.8 95.3%	90.7 95.5%	98.9 92.2%	88.3 93.8%	4.7 4.7%	4.3 4.5%	8.4 7.8%	5.8 6.2%

Number of reads was calculated from two biological replicates.

**Table 2:** Genes ID, annotation, functional category and Log_2_ FC of DE genes unique to drought-resistant genotypes (DR1&DR2) at 1h PEG post-treatment. NA means not annotated, NC means not categorized. The functional category was extracted from MapMan analysis using the list of DE genes that show Log_2_ FC ≥ 2 or ≤ −2 and *q*-value < 0.05. Down-regulated genes are indicated in red.

Gene ID	Annotation	Functional Category	Log_2_FC	
			DR1	DR2
*SOBIC.001G319700*	Glutathione S-transferase, putative	Antioxidant/detoxification	9.39	3.34
*SOBIC.005G112400*	Cysteine proteinase EP-B 1 precursor	Protein degradation	8.01	5.76
*SOBIC.001G114800*	NA	NC	7.67	3.19
*SOBIC.005G169300*	WIP5 - Wound-induced protein precursor	Stress (biotic)	7.03	7.17
*SOBIC.002G063800*	Transferase family protein, putative	Stress (biotic)	6.84	2.65
*SOBIC.003G425900*	Expressed protein	NC	6.43	2.51
*SOBIC.003G063100*	NA	NC	5.91	6.76
*SOBIC.009G159500*	PSF1 - Putative GINS complex subunit	NC	5.87	4.58
*SOBIC.010G041900*	Dehydrin, putative, expressed	NC	5.79	2.93
*SOBIC.007G029800*	LTPL24 - Protease inhibitor/seed storage/LTP protein precursor, expressed	NC	5.33	9.45
*SOBIC.003G313800*	Cytokinin dehydrogenase precursor	Hormone metabolism	5.13	2.15
*SOBIC.006G190100*	Naringenin, 2-oxoglutarate 3-dioxygenase	2nd metabolism	5.05	2.96
*SOBIC.001G058800*	Cysteine proteinase EP-B 1 precursor, putative	Protein degradation	5.02	7.40
*SOBIC.002G251000*	Expressed protein	NC	5.00	2.57
*SOBIC.004G309000*	Alpha-amylase precursor	Carbohydrate Digestion	4.83	3.83
*SOBIC.002G324000*	Isocitrate lyase, putative	Glycolysis	4.82	4.36
*SOBIC.010G166800*	Amino acid transporter	Amino acid transport	4.63	2.77
*SOBIC.004G249950*	NA	NC	4.56	2.13
*SOBIC.003G400400*	Cysteine proteinase inhibitor precursor protein	Protein degradation	4.55	2.48
*SOBIC.003G395000*	Cysteine proteinase EP-B 1 precursor, putative	Protein degradation	4.48	5.03
*SOBIC.001G344300*	NA	NC	4.40	4.60
*SOBIC.005G009600*	Pectinesterase	NC	4.01	7.11
*SOBIC.001G485200*	AP2 domain containing protein	Transcription factor	4.01	2.31
*SOBIC.004G133600*	Nodulin mtn3 family protein, putative	NC	3.84	4.46
*SOBIC.003G079200*	Transposon protein, putative, unclassified	NC	3.53	2.43
*SOBIC.002G260100*	NA	NC	3.39	2.21
*SOBIC.005G099000*	Glycosyl hydrolase, putative	Stress (biotic)	3.34	2.11
*SOBIC.002G269032*	Dehydration-responsive element (DREB1B-like) proteinputaexpressed	Transcription factor	3.33	2.40
*SOBIC.003G282901*	DUF584 domain containing protein	NC	3.05	2.90
*SOBIC.001G401200*	Pathogenesis-related Bet v I family protein	NC	2.97	2.43
*SOBIC.004G273300*	Ubiquitin family protein, putative	Protein degradation	2.90	2.00
*SOBIC.004G315200*	Expressed protein	NC	2.83	2.21
*SOBIC.004G147900*	Cupin domain containing protein	Stress (abioticv)	2.82	2.26
*SOBIC.002G269100*	Similar to DREB1a-like protein	Transcription factor	2.78	3.41
*SOBIC.001G443800*	Glutamate decarboxylase, putative	Amino acid metabolism	2.78	2.01
*SOBIC.003G430400*	Oscml31 - Calmodulin-related calcium sensor proteinexpressed	NC	2.76	2.00
*SOBIC.002G139400*	Expressed protein	Hormone metabolism	2.76	2.49
*SOBIC.002G416600*	Peroxidase precursor	Antioxidant/detoxification	2.72	2.10
*SOBIC.002G415600*	AP2 domain containing protein	Transcription factor	2.67	2.28
*SOBIC.009G217600*	STE_MEKK_ste11_MAP3K.19 - STE kinases incluhomologs to sterile 7, sterile 11 and sterile 20 from yeast, expressed	Posttransilation modificati.	2.66	2.12
*SOBIC.001G398500*	Long cell-linked locus protein	NC	2.61	2.70
*SOBIC.005G016600*	DUF567 domain containing protein	NC	2.58	2.30
*SOBIC.009G071800*	6-phosphofructokinase	Glycolysis	2.55	2.52
*SOBIC.009G043700*	Glutathione S-transferase	Antioxidant/detoxification	2.49	2.16
*SOBIC.002G250900*	Expressed protein	NC	2.35	2.95
*SOBIC.003G361100*	Hs1, putative	NC	2.33	2.18
*SOBIC.003G264500*	Glutathione S-transferase, putative	Antioxidant/detoxification	2.25	2.19
*SOBIC.001G319600*	Glutathione S-transferase	Antioxidant/detoxification	2.22	2.38
*SOBIC.005G163800*	Laccase precursor protein	2nd metabolism	−2.01	−2.12
*SOBIC.004G299600*	Transposon protein, putative	NC	−2.07	−2.16
*SOBIC.009G250200*	Transmembrane amino acid transporter proteinputatexpressed	Amino acid transport	−2.37	−2.07
*SOBIC.008G036800*	Chalcone synthase	2nd metabolism	−2.38	−2.21
*SOBIC.001G082700*	TKL_IRAK_crrlk1l-1.1 - The crrlk1l-1 subfamily hahomology to the crrlk1l homolog, expressed	Signaling	−2.39	−2.34
*SOBIC.003G348300*	Expressed protein	NC	−2.47	−2.12
*SOBIC.004G260600*	Fatty acid desaturase	Lipid metabolism	−2.47	−2.45
*SOBIC.009G096300*	ATOFP17/OFP17	NC	−2.48	−2.67
*SOBIC.003G278100*	Expressed protein	NC	−3.19	−2.87
*SOBIC.002G071600*	AP2 domain containing protein	Transcription factor	−3.55	−2.20
*SOBIC.008G177400*	Strictosidine synthase	2nd metabolism	−4.20	−4.51

**Table 3:** Genes ID, annotation, functional category and Log_2_ FC of DEG in drought-resistant genotypes treated with PEG for 6 h. NA means not annotated, NC means not categorized. The functional category was extracted from MapMan analysis using the list of DE genes that show Log_2_ FC ≥ 2 or ≤ 2 and *q*-value < 0.05. Down-regulated genes are indicated in red.

Gene ID	Annotation	Functional Category	Log_2_FC	
			DR1	DR2
*SOBIC.009G025500*	Osrci2-5 -low temperature and salt responsive protein	NC	9.63	6.75
*SOBIC.003G295600*	DUF1264 domain containing protein	NC	9.36	7.53
*SOBIC.001G311200*	NA	NC	9.00	6.78
*SOBIC.001G381700*	NA	NC	8.95	6.77
*SOBIC.002G151200*	LEA 4, seed maturation protein	Development	8.68	7.02
*SOBIC.003G372600*	NA	NC	8.62	4.47
*SOBIC.004G114600*	NA	NC	8.03	6.70
*SOBIC.001G090900*	LEA, late embryogenesis abundant protein D-34	Development	7.72	6.19
*SOBIC.004G195900*	LEA, late embryogenesis abundant protein D-34	Development	7.40	7.73
*SOBIC.001G177600*	Osfbx103 - F-box domain containing protein	NC	7.32	7.44
*SOBIC.010G016500*	Dehydration-responsive element-binding protein	Transcription factor	7.24	4.10
*SOBIC.001G425900*	Edm2	Transcription factor	7.15	7.94
*SOBIC.001G091000*	LEA, late embryogenesis abundant protein D-34	Development	6.70	4.52
*SOBIC.001G208500*	Aquaporin protein, putative, expressed	Transport/major proteins	5.91	5.50
*SOBIC.007G156700*	AP2 domain containing protein, expressed	Transcription factor	5.82	6.33
*SOBIC.009G037100*	Oxidoreductase	Misc. (oxidoreductase)	5.57	4.81
*SOBIC.003G198100*	NA	NC	5.47	3.17
*SOBIC.004G092800*	Hsp20/alpha crystallin family protein	NC	5.45	2.76
*SOBIC.004G196000*	Late embryogenesis abundant protein D-34	Development	5.45	3.18
*SOBIC.008G040600*	Bzip transcription factor domain containing protein	Transcription factor	5.38	4.05
*SOBIC.004G185000*	STRUBBELIG-RECEPTOR FAMILY 7 precursor	Protein/post-translation modification	5.31	3.73
*SOBIC.003G241400*	Seed maturation protein PM41	NC	5.13	3.08
*SOBIC.002G391100*	Peroxiredoxin	Redox/oxidation/reduction	4.91	4.72
*SOBIC.001G149300*	Expressed protein	NC	4.71	2.82
*SOBIC.001G497700*	Late embryogenesis abundant protein D-34	Development	4.61	3.94
*SOBIC.007G138000*	Zinc finger A20 and AN1 domain-containing stress-associated protein	Transcription factor	4.59	3.65
*SOBIC.006G210600*	NA	NC	4.56	2.43
*SOBIC.001G342500*	PDI	NC	4.33	3.80
*SOBIC.003G268800*	STE_MEKK_ste11_MAP3K.5-STE kinases	Protein/post-translation modification	4.30	2.20
*SOBIC.004G221900*	DUF584 domain containing protein	NC	4.24	2.58
*SOBIC.008G050600*	Ethylene-responsive transcription factor ERF114	Transcription factor	4.14	2.58
*SOBIC.003G429401*	NA	NC	4.04	2.51
*SOBIC.005G003200*	Asparagine synthetase	Amino acid metabolism	3.98	2.33
*SOBIC.003G270200*	Dehydrin family protein	NC	3.97	2.96
*SOBIC.003G156200*	Cytochrome P450	Misc. (Cytochrome P450)	3.94	5.12
*SOBIC.003G312300*	Homocysteine S-methyltransferase protein	Amino acid metabolism	3.85	3.00
*SOBIC.009G230000*	Transcription factor HBP-1b	Transcription factor	3.72	2.79
*SOBIC.002G117100*	Cyclin-dependent kinase G-1	Protein/post-translation modification	3.67	2.28
*SOBIC.003G442601*	Transcription initiation factor IIA gamma chain	Transcription factor	3.63	2.21
*SOBIC.003G082500*	Hsp20/alpha crystallin family protein	Stress/abiotic	3.62	6.29
*SOBIC.003G293500*	Heat shock protein 101	Stress/abiotic	3.57	2.46
*SOBIC.002G308900*	Ubiquitin fusion protein	Protein/degradation	3.49	-2.46
*SOBIC.005G016400*	DUF567 domain containing protein	NC	3.46	2.49
*SOBIC.010G276100*	Transporter family protein	Transport/Sugar	3.42	3.05
*SOBIC.001G391300*	Glycosyl transferase 8 domain containing protein	Carbohydrate metabolism	3.37	2.18
*SOBIC.001G035000*	ZOS3-22-C2H2 zinc finger protein	NC	3.33	2.58
*SOBIC.001G027800*	Osman05-Endo-Beta-Mannanase	Cell wall modification	3.31	2.09
*SOBIC.001G114800*	NA	NC	3.27	4.11
*SOBIC.009G054500*	Lipase	Lipid metabolism	3.19	2.03
*SOBIC.001G044300*	Transcription factor	Transcription factor	3.14	3.41
*SOBIC.006G025066*	Lysm domain containing protein	NC	3.11	7.10
*SOBIC.004G286600*	Dehydrin	NC	3.02	2.41
*SOBIC.006G119600*	GRAM and C2 domains containing protein	Protein/degradation	2.98	4.04
*SOBIC.001G128900*	Cytochrome P450	Misc. (Cytochrome P450)	2.98	3.16
*SOBIC.003G369100*	AP2 domain containing protein	NC	2.96	2.39
*SOBIC.010G045100*	Osftl2 FT-Like2 homologous to Flowering Locus T	Development	2.93	6.35
*SOBIC.010G088500*	Phosphate-induced protein 1	Signaling	2.89	2.84
*SOBIC.005G085400*	DNA-directed RNA polymerase subunit beta	NC	2.87	2.60
*SOBIC.001G147500*	Cytochrome P450	Misc. (Cytochrome P450)	2.83	2.03
*SOBIC.001G162700*	Endonuclease/exonuclease/phosphatase family	Chromatic/DNA structure	2.80	2.23
*SOBIC.008G109600*	Oxidoreductase, 2OG-Fe oxygenase family protein	Secondary metabolism	2.69	2.13
*SOBIC.001G068600*	App1	NC	2.68	2.60
*SOBIC.001G509300*	NA	NC	2.66	2.24
*SOBIC.001G174100*	GRAS family transcription factor containing protein	Transcription factor	2.60	2.24
*SOBIC.004G321800*	Saccharopine dehydrogenase	Amino acid metabolism	2.58	2.09
*SOBIC.002G247900*	Hypothetical protein	NC	2.50	3.27
*SOBIC.004G192800*	Mitochondrial prohibitin complex protein 1	Stress/abiotic	2.39	2.39
*SOBIC.009G080400*	Hydroquinone glucosyltransferase	Secondary metabolism	2.38	2.33
*SOBIC.006G097800*	Anthocyanin 5-O-glucosyltransferase	Hormone metabolism	2.37	2.01
*SOBIC.004G088551*	NA	NC	2.37	2.11
*SOBIC.002G393100*	Dehydrogenase/reductase	Misc. (dehydrogenase/reductase)	2.32	2.42
*SOBIC.007G108800*	NA	NC	2.29	2.70
*SOBIC.002G089600*	DEF8 - Defensin and Defensin-like DEFL family	NC	2.28	2.35
*SOBIC.001G170301*	GASR2 - Gibberellin-regulated GASA/GAST/Snakin	Hormone Metabolism	2.24	2.14
*SOBIC.005G103600*	NA	NC	2.17	2.12
*SOBIC.009G171400*	Oxidoreductase, aldo/keto reductase family protein	Carbohydrate metabolism	2.12	2.28
*SOBIC.001G482800*	ZIM domain containing protein	NC	2.07	2.16
*SOBIC.002G043400*	Erythrocyte binding protein	NC	2.04	2.35
*SOBIC.009G080300*	Uncharacterized protein KIAA1310	NC	−2.00	−2.19
*SOBIC.006G028000*	Amidase	Misc. (nitrile lyase)	−2.05	−2.72
*SOBIC.004G110200*	Zinc finger, C3HC4 type domain containing protein	Transcription factor	−2.12	−2.56
*SOBIC.002G115600*	Membrane protein	NC	−2.16	−2.29
*SOBIC.001G232200*	Glycine-rich cell wall structural protein 2 precursor	Cell wall	−2.21	−6.49
*SOBIC.005G163800*	Laccase precursor protein	Secondary metabolism	−2.23	−3.17
*SOBIC.002G286400*	Uncharacterized protein PA4923	Amino acid metabolism	−2.31	−2.10
*SOBIC.002G063700*	NB-ARC domain containing protein	Stress/biotic	−2.35	−3.04
*SOBIC.008G158400*	LTPL116 - Protease inhibitor/seed storage protein	Misc. (seed storage protein)	−2.40	−5.38
*SOBIC.002G163200*	NA	NC	−2.45	−3.11
*SOBIC.008G158600*	LTPL116 - Protease inhibitor/seed storage protein	Misc. (seed storage protein)	−2.47	−2.64
*SOBIC.008G162700*	Fatty acid hydroxylase	Lipid metabolism	−2.51	−2.00
*SOBIC.005G195400*	Helix-loop-helix DNA-binding domain containing-P	Transcription factor	−2.66	−2.47
*SOBIC.002G286566*	Carrier	NC	−2.67	−2.30
*SOBIC.008G158501*	LTPL116 - Protease inhibitor/seed storage protein	Misc. (seed storage protein)	−2.72	−3.93
*SOBIC.005G086600*	O-methyltransferase	Secondary metabolism	−2.75	−3.16
*SOBIC.004G180700*	NA	Misc. (oxidase/copper)	−2.77	−2.52
*SOBIC.001G317800*	Glutathione S-transferase	Misc. (Oxidoreductase)	−2.91	−2.94
*SOBIC.008G158266*	NA	NC	−3.03	−6.59
*SOBIC.005G002700*	Fatty acid desaturase, putative	Lipid metabolism	−3.11	−2.11
*SOBIC.008G036800*	Chalcone synthase	Secondary metabolism	−3.15	−2.35
*SOBIC.003G278100*	Expressed protein	NC	−3.20	−2.45
*SOBIC.001G232500*	Glycine-rich cell wall structural protein 2 precursor	Cell wall	−3.23	−3.79
*SOBIC.004G260600*	Fatty acid desaturase	Lipid metabolism	−3.26	−3.19
*SOBIC.010G156500*	NA	NC	−3.29	−3.99
*SOBIC.006G096100*	Oxidoreductase, aldo/keto reductase family protein	Secondary metabolism	−3.58	−8.28
*SOBIC.004G228600*	NA	NC	−3.78	−8.33
*SOBIC.005G215000*	Cytochrome P450	Misc. (Cytochrome P450)	−3.80	−2.69
*SOBIC.003G059900*	Expansin precursor	Cell wall modification	−3.82	−2.42
*SOBIC.008G158900*	Chitinase 2	NC	−3.88	−2.63
*SOBIC.002G087200*	Osfbx231 - F-box domain containing protein	NC	−3.99	−6.20
*SOBIC.001G502000*	Inorganic phosphate transporter	Transport/Phosphate	−4.05	−2.21
*SOBIC.007G059200*	Transferase family protein	Secondary metabolism	−4.62	2.54
*SOBIC.009G158500*	NA	Signaling	−4.75	−2.79
*SOBIC.010G044001*	NA	NC	−6.16	−3.26
*SOBIC.008G158332*	LTPL116 - Protease inhibitor/seed storage protein	Misc. (seed storage protein)	−6.38	−5.31
*SOBIC.002G228800*	Expressed protein	NC	−6.63	−7.25
*SOBIC.001G403350*	NA	NC	−6.91	−2.68
*SOBIC.009G244600*	MYB family transcription factor	RNA/Transcription	−7.01	−8.60
*SOBIC.003G437900*	Decarboxylase	Amino acid metabolism	−7.50	−5.70

## Supplementary Materials

Supplementary materials can be found at https://www.mdpi.com/1422-0067/21/3/772/s1.

## Abbreviations

DE	Differentially expressed
