# Supplementary material for: Transcriptome Analysis of Drought-Resistant and Drought-Sensitive Sorghum (Sorghum bicolor) Genotypes in Response to PEG-Induced Drought Stress
Source: Int J Mol Sci. 2020 Jan 24;21(3):772. doi: 10.3390/ijms21030772 (PMC7037816; doi:10.3390/ijms21030772)
Supplement: Supplementary file 1 [file ijms-21-00772-s001.zip › Suppl Figure S1 MapMan.pptx]

## Slide 1
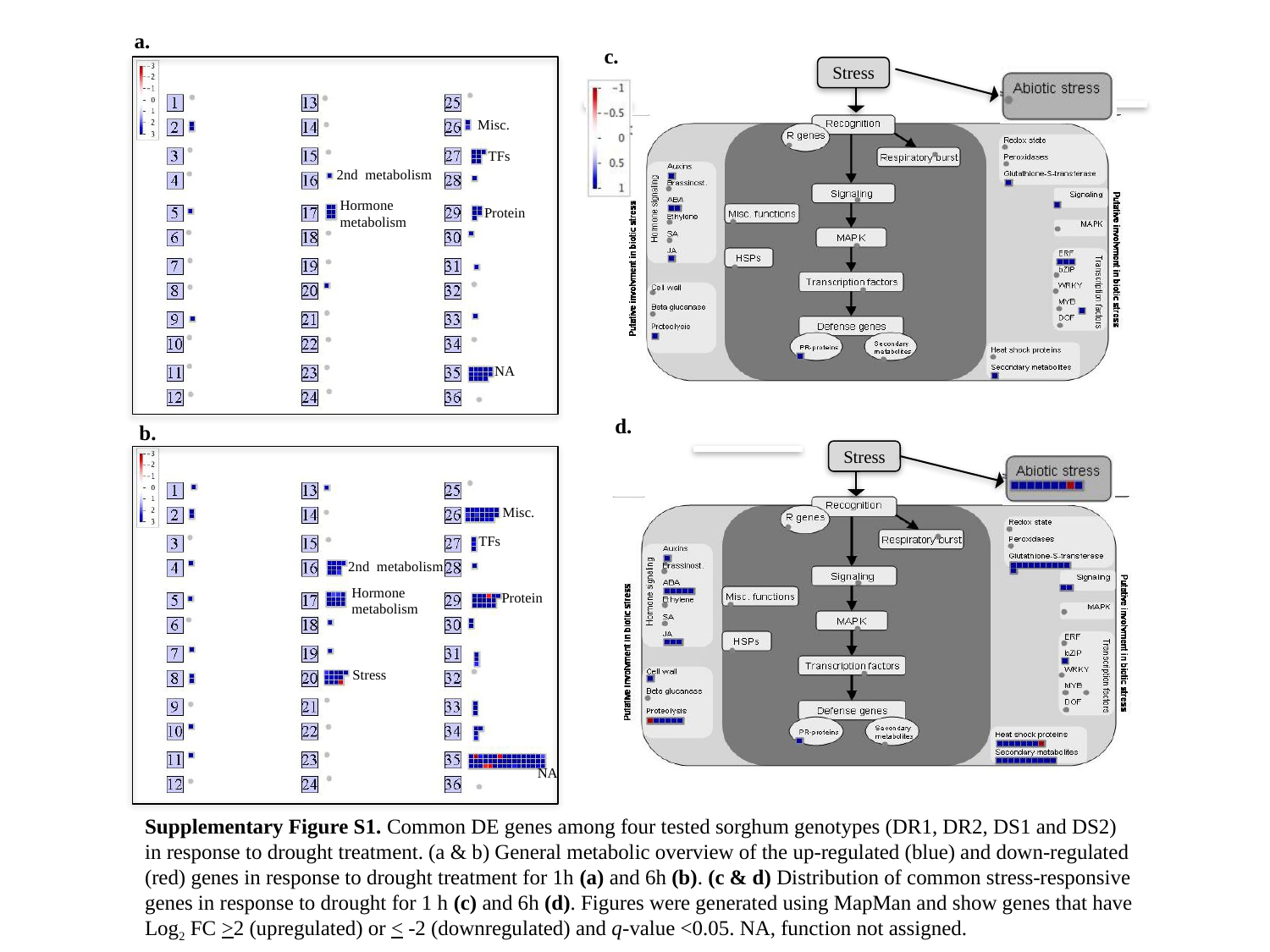

a.
Misc.
TFs
2nd metabolism
Hormone
metabolism
Protein
NA
c.
Stress
d.
b.
Misc.
TFs
2nd metabolism
Hormone
metabolism
Protein
Stress
NA
Stress
Supplementary Figure S1. Common DE genes among four tested sorghum genotypes (DR1, DR2, DS1 and DS2) in response to drought treatment. (a & b) General metabolic overview of the up-regulated (blue) and down-regulated (red) genes in response to drought treatment for 1h (a) and 6h (b). (c & d) Distribution of common stress-responsive genes in response to drought for 1 h (c) and 6h (d). Figures were generated using MapMan and show genes that have Log2 FC >2 (upregulated) or < -2 (downregulated) and q-value <0.05. NA, function not assigned.
