# Supplementary material for: Transcriptome Analysis of Drought-Resistant and Drought-Sensitive Sorghum (Sorghum bicolor) Genotypes in Response to PEG-Induced Drought Stress
Source: Int J Mol Sci. 2020 Jan 24;21(3):772. doi: 10.3390/ijms21030772 (PMC7037816; doi:10.3390/ijms21030772)
Supplement: Supplementary file 1 [file ijms-21-00772-s001.zip › Supplementary Figure S2.pptx]

## Slide 1
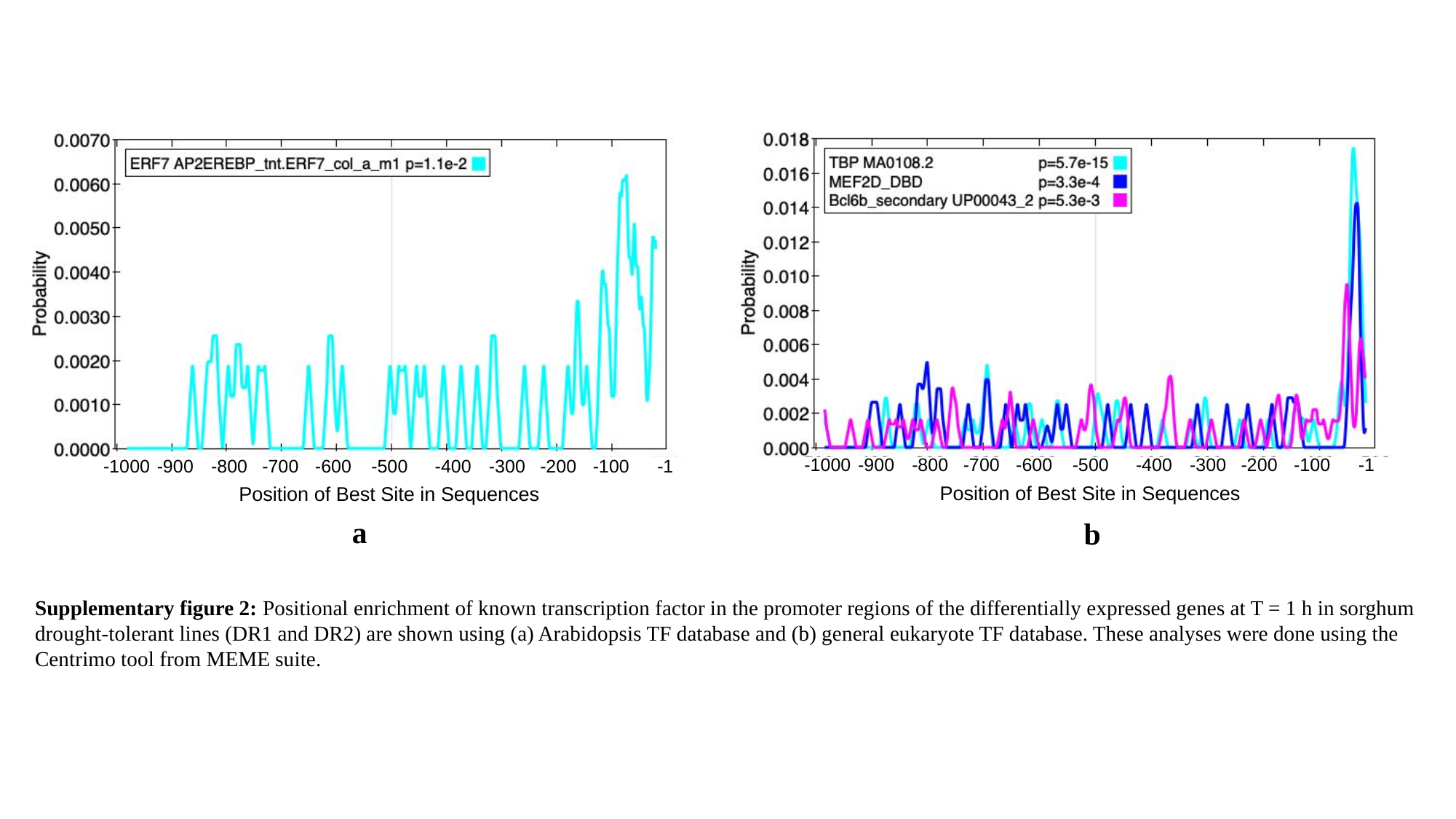

-1000
-900
-800
-700
-600
-500
-400
-300
-200
-100
-1
Position of Best Site in Sequences
b
-1000
-900
-800
-700
-600
-500
-400
-300
-200
-100
-1
Position of Best Site in Sequences
a
Supplementary figure 2: Positional enrichment of known transcription factor in the promoter regions of the differentially expressed genes at T = 1 h in sorghum drought-tolerant lines (DR1 and DR2) are shown using (a) Arabidopsis TF database and (b) general eukaryote TF database. These analyses were done using the Centrimo tool from MEME suite.
